# Impact of a Risk Management Plan on *Legionella* Contamination of Dental Unit Water

**DOI:** 10.3390/ijerph120302344

**Published:** 2015-02-23

**Authors:** Erica Leoni, Laura Dallolio, Francesca Stagni, Tiziana Sanna, Giovanni D’Alessandro, Gabriela Piana

**Affiliations:** 1 Department of Biomedical and Neuromotor Sciences, Unit of Hygiene, Public Health and Medical Statistics, University of Bologna, via San Giacomo 12, 40126, Bologna, Italy; E-Mail: laura.dallolio@unibo.it; 2 Department of Biomedical and Neuromotor Sciences, Unit of Odontostomatological Sciences, University of Bologna, via San Vitale 59, 40125, Bologna, Italy; E-Mails: francesca.stagni6@unibo.it (F.S.); dr.dalessandro@gmail.com (G.D.A.); gabriela.piana@unibo.it (G.P.); 3 Department of Biomedical and Neuromotor Sciences, School of Hygiene and Preventive Medicine, University of Bologna, via San Giacomo 12, 40126, Bologna, Italy; E-Mail: tiziana.sanna@studio.unibo.it

**Keywords:** *Legionella* spp., dental unit waterlines, dental unit disinfection, dental unit water safety plan

## Abstract

The study aimed to assess the prevalence of *Legionella* spp. in dental unit waterlines of a dental clinic and to verify whether the microbiological parameters used as indicators of water quality were correlated with *Legionella* contamination. A risk management plan was subsequently implemented in the dental health care setting, in order to verify whether the adopted disinfection protocols were effective in preventing *Legionella* colonization. The water delivered from syringes and turbines of 63 dental units operating in a dental clinic, was monitored for counts of the heterotrophic bacteria *P. aeruginosa* and *Legionella* spp. (22 °C and 37 °C). At baseline, output water from dental units continuously treated with disinfection products was more compliant with the recommended standards than untreated and periodically treated water. However, continuous disinfection was still not able to prevent contamination by *Legionella* and *P. aeruginosa*. *Legionella* was isolated from 36.4%, 24.3% and 53.3% of samples from untreated, periodically and continuously treated waterlines, respectively. The standard microbiological parameters used as indicators of water quality proved to be unreliable as predictors of the presence of *Legionella*, whose source was identified as the tap water used to supply the dental units. The adoption of control measures, including the use of deionized water in supplying the dental unit waterlines and the application of a combined protocol of continuous and periodic disinfection, with different active products for the different devices, resulted in good control of *Legionella* contamination. The efficacy of the measures adopted was mainly linked to the strict adherence to the planned protocols, which placed particular stress on staff training and ongoing environmental monitoring.

## 1. Introduction

*Legionella* spp. are waterborne pathogen bacteria that are frequently isolated from man-made aquatic environments such as hot water plants, shower heads, cooling towers, spas, whirlpools, humidifiers and evaporative condensers. *Legionellae* may also colonize dental unit waterlines, *i.e.* the narrow-bore plastic tubing that carries water to the high-speed handpieces and air/water syringes of dental chairs. Therefore, the output water from dental units may be a potential source of infection for both dental health care personnel and patients. The presence of bacteria within the waterlines is conducive to the formation of biofilms, which protect the organisms from desiccation, chemical insult and predation. Moreover, the microorganisms on the surfaces are continuously released from the biofilm into the water flowing through or standing in the tubing lumen, so that biofilm becomes the primary reservoir for continued contamination of the system [1,2,3,4,5,6].

*Legionella* is frequently detected in the output water of dental devices [7,8,9,10,11,12,13]. Although isolation of *Legionella* is frequently reported, particularly where there are multiple chairs (e.g., dental schools), only a small number of case-reports of *Legionella* infection directly linked to contaminated dental waterlines have been described. A fatal case of pneumonia due to *Legionella* was reported in an 82–year-old Italian woman exposed to a dental unit contaminated by *L. pneumophila* SG1. Molecular typing confirmed clonal relation between the clinical and environmental strains [14]. In another report, sub-clinical infection with *L. pneumophila* was related to exposure to aerosol generated by the dental handpieces in a dental school environment [15]. Finally, there is evidence that students and employees at dental clinics had a significantly higher prevalence of *Legionella* seropositivity, compared to controls [16,17,18,19,20].

Despite the lack of documented adverse health effects in immunocompetent persons, the presence of *Legionella* spp. in dental unit waterlines is cause for concern due to the growing number of vulnerable individuals (e.g., the elderly, immune-compromised patients). Furthermore, exposing patients or dental teams to contaminated water is not consistent with universally accepted infection-control principles [21]. The American Dental Association (ADA) has set a heterotrophic bacteria load of ≤200 cfu/mL for water delivered from dental unit waterlines [22], while the CDC guidelines for Infection Control in Dental Health-Care Settings suggest that the numbers of microorganisms should meet nationally recognized standards for safe drinking water, considering that the standard set for the USA by the Environmental Protection Agency (EPA) is ≤500 cfu/mL [21]. In Italy standards for drinking water include: ≤100 cfu/mL of heterotrophic bacteria (HPC at 22 °C) and ≤20 cfu/mL of mesophilic bacteria (HPC at 37 °C), in accordance with the European and national directives [23,24]. In order to achieve these recommended values, measures for the control of microbial contamination in dental unit waterlines are required, as suggested by various guidelines [21,25]. In the Italian guidelines for the control and prevention of legionellosis established by the Italian Health Ministry [26], now being updated, the dental health care setting is included among health care facilities, suggesting an interactive approach based on a risk assessment plan in order to control *Legionella* contamination. In dental settings the integrated approach to risk management includes a set of technical-practical measures such as waterline flushing, independent water reservoir systems, deionized or sterilized water, inline micro pore filtration, and periodic or continuous chemical disinfection.

The need to supply dental unit waterlines with disinfection systems, in order to minimize microbial contamination and biofilm formation, has already been widely discussed [27,28,29,30,31]. The efficacy of different chemical treatments and disinfection protocols, applied both continuously [28,32,33,34,35] or intermittently [28,34,36,37,38] has been evaluated for use in dental units. On the basis of these studies and the findings of a recent review by O’Donnell *et al.* [3], the differences observed are not so much linked to the active product used, but rather to the type of protocol applied (continuous or intermittent) and, above all, adherence to disinfection protocols by dental personnel.

The aim of this study was firstly to assess the prevalence of *Legionella* spp. in a dental health care setting and to verify whether the microbiological parameters used as indicators of drinking water quality were correlated with the *Legionella* contamination of dental unit waterlines, thus proving to be reliable predictors of the presence of *Legionella*. A second aim was to measure the impact of a risk management plan on *Legionella* contamination of dental unit water and to evaluate its effectiveness in preventing *Legionella* colonization of dental unit waterlines.

## 2. Materials and Methods

### 2.1. Setting

The investigation was carried out in a university odonto-stomatological clinic housing nine divisions and 63 operating spaces which are used for public dental treatment as well as for teaching students of Orthodontics and Dental Prosthesis, and Dental Hygiene. The clinic occupies two floors of a 4-sided, 4-storey building; a south-facing secondary wing extends from the western side of the main building (a map of the dental clinic is provided as Supplementary Material). The whole building is supplied by a single water system which has been modified over time. The various divisions are situated on the ground and first floors. Each operating space is a single room where one dental unit is in use, making a total of 63 devices (Table 1).

The units vary in manufacture and age (ranging from 1998 to 2013). The six oldest dental units (manufactured between 1998–2001 by Castellini, Imola, Italy) and five of the newer ones (manufactured between 2007–2008 by Anthos, Imola, Italy) are fed directly from the municipal water network and have no independent supply system; these units cannot therefore be treated with demineralized and disinfected water, either intermittently or continuously. Another 37 dental units (Castellini, from 2004 to 2010) can be supplied directly from the water mains or through an independent system that receives water from a 1 liter polypropylene bottle to which a disinfectant product can be added for continuous treatment. These dental units also allow the dental care worker to activate a periodic automated disinfection cycle with a disinfectant product, which generates peracetic acid, peracetyl ions and hydrogen peroxide equivalent to 0.26% of peracetic acid (Rely+On^TM^ Peracilyse, by Antec International Limited, Sudbury, UK). The disinfection system can be applied between one patient and the next (intermittent), or for disinfection at the end of the day (periodic). The remaining 15 dental units take their water from a 2 liter polyethylene bottle which, in accordance with the directions of the manufacturer, is filled with tap water to which a disinfectant agent is added. For 11 of these units (A-dec, Newberg, OR, USA) an oxidant product is used, based on sodium percarbonate (use concentration 0.05%–0.15%), cationic surfactants (0.01%–0.03%) and silver nitrate (0.001%–0.005%) (ICX^®^ by A-dec, Inc.). The other four dental units (Eurodent, Bologna, Italy) use a disinfection product (Calbenium^®^ by Ariel West Inc. Hallandale, FL, USA) containing chloramine and benzalkonium chloride at a use concentration of 2%.

**Table 1:** Location of the divisions in the clinic and number of dental units treated with different types of disinfection, at baseline.

Location	Division	Number of Operating Spaces	Number of Dental Units		
			No Disinfection treatment	Periodic Disinfection (Rely + OnTM Peracilyse)	Continuous Disinfection (ICX® or Calbenium®)
Ground floor Main building	Disabled	3		3	
	First Visit	5		5	
Ground floor Secondary wing	Conservative	7	4	3	
	Orthodontics	4	4		
First floor Main building	Endodontrics	6	2	3	1
	Surgery	4		3	1
	Periodontics	7	1		6
	Prosthesis	7			7
First floor Secondary wing	Dental school area	20		20	
		63	11	37	15

At baseline, the microbiological quality of the output water dispensed by the 63 units was monitored over a period of about three months (from April to June 2013) under normal use conditions. In this phase, the output water from each dental unit was sampled and tested once. Since some dental units resulted contaminated by *P. aeruginosa* and *Legionella* spp., a risk management plan was developed and implemented in order to identify the specific corrective measures to be taken to remove the contamination and to control the re-colonization of the dental units. For a period of about eight months (from September 2013 to April 2015), the efficacy of the measures adopted was assessed by carrying out microbiological tests repeated after each specific intervention (for example the shock treatment of the contaminated units), up to the final definition of the water safety plan. The post intervention follow-up was carried out by monitoring the 63 units over a period of about three months (from May to July 2014). Also in this final monitoring, the output water dispensed by each dental unit was sampled and tested once.

### 2.2. Control Program and Water Safety Plan

Table 2 shows the main aspects considered in the internal quality plan and the identification of actions for prevention and control. The control program included daily management measures (supply water, flushing) and periodic maintenance of dental units, and a water safety plan, which introduced a disinfection procedure for the dental unit waterlines. The procedure included both a continuous disinfection treatment with low dosage products and a periodic treatment with a higher dosage of active agents, in accordance with the suggestions of the different dental unit manufacturers. In addition, a shock treatment was recommended in case of contamination by *Legionella* spp. Table 3 describes the main ordinary and extraordinary procedures implemented in the dental clinic, and how frequently to do them.

**Table 2:** Main steps in the definition of the internal quality plan for the control of *Legionella* contamination in dental health care setting.

Establishment of a work group	Health director, head of the prevention and protection service, employees responsible for divisions, manager for technical systems and dental units maintenance
Risk assessment	Past history of the facility (previous cases of legionellosis)
	Environmental factors (supply water)
	Factors linked to dental units (age, presence of disinfection systems, maintenance) and dental practices (frequency of use, invasive procedures)
	Type of patients under care
Identification of control measures	Definition of modalities and frequency of dental units maintenance and recording of ordinary and extraordinary maintenance works
	Identification of most appropriate decontamination methods, in accordance with manufacturers and reference to the literature and guidelines
	Review of the list of manufacturers and companies supplying disinfection systems, to contact them quickly if necessary
Environmental monitoring	Planning of environmental checks of supply water and water delivered by dental units
Training and communication	Organization of training for staff

### 2.3. Processing of Water Samples

Water samples were collected from the distal outlets of the air/water syringes and turbines at the beginning of the work day. In addition, samples of water from the mains were collected from the faucets of each division. Before taking the samples, water was flushed for two minutes. In order to neutralize the residual disinfectant, 10% sodium thiosulphate was added to the sterile bottles for bacteriological analysis (1 ml/L). The samples were kept at 4 °C and analyzed within 3–5 h of sampling.

**Table 3:** Preventive and corrective measures implemented in the internal quality plan for the control of *Legionella* contamination in the dental health care setting.

Control measures and management of the dental units		Use of deionised water instead of mains water in supplying the dental unit waterlines.
		Application of a protocol of continuous and periodic disinfecting treatment of dental unit waterlines
		Allow the outlet water from syringes and turbines to run for several minutes at least once a day if dental unit is not used. Flush out for 20-30 seconds after each patient and for several minutes before the daily start of the clinic work (CDC, 2003)
		Regular microbiological monitoring of the waterlines (at least once a year)
		When necessary apply chemical shock to the dental unit waterlines (on the basis of microbiological monitoring)
		Record any maintenance work (ordinary and extraordinary) and results of monitoring. Check that the all the control measures are implemented
Protocols of treatment introduced for the dental units with independent water supply systems	by Castellini Company	Continuous disinfection with hydrogen peroxide (concentration: 0.06%)
		Daily cycle of treatment with a disinfectant product generating peracetic acid, peracetyl ions and hydrogen peroxide equivalent to 0.26% of peracetic acid (Rely+On Peracilyse): the product is put inside the external dental unit bottle at the end of the clinic day, left for 10 minutes and then rinsed out
	by A-dec Company	Continuous disinfection with ICX^®^ (use concentration: 0.01%)
		Weekly cycle of treatment with an alkaline based peroxide agent (Sterilex Ultra, Sterilex Corporation, Maryland, USA) used at concentration of 0.5%: the product is put inside the external dental unit bottle at the end of the clinic day, and left overnight; the solution is then rinsed out in the morning
	by Eurodent Company	Continuous disinfection with Calbenium^®^ (use concentration: 2%).
Corrective measures in case of contamination		Shock treatment (dental units with independent water supply system):
		Sterilize the supply bottle and suction needle
		Add 300 ml of hydrogen peroxide 3% to the bottle
		Activate the dental unit and press the water button 4-5 times allowing the hydrogen peroxide to exit
		Leave it to rest for 10 minutes
		Remove the bottle with the residual hydrogen peroxide and replace it with another sterile bottle containing hydrogen peroxide 0.06%
		Let the water run from the instrument to rinse the waterlines
		Check the results both immediately after decontamination and periodically to verify the efficacy of the adopted measures

The following process indicators were determined: Heterotrophic Plate Count at 22 °C (HPC 22 °C), Heterotrophic Plate Count at 37 °C (HPC 37 °C), and *Pseudomonas aeruginosa.* The HPCs were performed by pour plate method on Plate Count Agar (Biolife, Milan, Italy) at 37 °C and 22 °C, for 48 and 72 h respectively (UNI EN ISO 6222:2001) and the standard membrane filter technique was used to detect *P. aeruginosa* (UNI EN ISO 16266:2008, medium: *Pseudomonas* selective Agar—Biolife). A volume of 100 mL was filtered using 4.7 cm cellulose acetate filters (0.45 µm pore size—Merck Millipore, Darmstadt, Germany). After an incubation period at 37 °C for 48–72 h, suspected colonies were identified using miniaturized biochemical tests (API 20NE, bioMérieux, Marcy l’Etoile, France).

In addition, *Legionella* spp. were detected according to the ISO11731 standard technique (1998) by pouring 1 liter of water through a polyamid filter with 0.20 µm diameter pores (Sartorius AG, Goettingen, Germany). The concentrate was suspended in 10 mL of sample water and vortexed for 15 min. An aliquot of the concentrate was examined as such, another aliquot was subjected to decontamination treatment with heat at 50 °C for 30 min. Both the concentrated and decontaminated samples were plated on Legionella GVPC selective Agar (Oxoid, Basingstoke, UK) and incubated at 35 °C in microaerophilic conditions for 14 days. The isolates were identified on the basis of cultural and serological features, as previously described [39,40].

### 2.4. Statistical Analysis

The values of the microbial loads were converted into Log_10_ x to normalize the non normal distributions, and the results are presented as geometric means. For negative samples, the detection limit was used. Differences between the microbial loads detected in the different groups of dental units (differently treated) were tested using standard one-way analysis of variance (ANOVA). Unpaired t-test was used to compare the HPCs in samples positive or negative for *Legionella* spp. and *P. aeruginosa* respectively. Paired t test was used as significance test when comparing the bacterial counts of the same dental units at baseline and after the implementation of the management plan. A *p* value < 0.05 was considered statistically significant.

## 3. Results and Discussion

### 3.1. Baseline Monitoring

Table 4 shows the results obtained at baseline, in relation to the disinfection treatment of the dental unit waterlines. At baseline all units were fed with water from the public water supply. As far as the HPCs at 37 and 22 °C are concerned, the tap water was found to satisfy the required standards for drinking water, but two out of nine samples were contaminated by *L. pneumophila* SG1. Both samples had been taken from taps on the first floor, in operating spaces of the main building.

Overall, the output water from the dental units presented not compliant colony counts in 38.1% of samples, considering the Italian limit for HPC at 22 °C for drinking water, and in 66.7% of samples considering both the limits for HPCs at 22 and 37 °C. *P. aeruginosa* and *Legionella* spp. were detected respectively in 22.2% and 34.9% of the samples (*L. pneumophila* SG1: 25.4%, *L. anisa*: 9.5%), although the results varied depending on the type of disinfection treatment adopted (Table 4).

The water from the dental units not subjected to any treatment showed microbial loads higher than the values recommended by the ADA and those set by the Italian regulations for drinking water (HPC at 22 °C) in 36.4% of samples. *P. aeruginosa* was detected in one sample out of 11 (9.1%); *L. pneumophila* SG1 was detected in two samples (18.2%), both taken from operating spaces on the first floor of the main building, and *L. anisa* was found in the output water of two dental units (18.2%) in the operating spaces situated on the ground floor of the secondary wing of the building.

The pattern of contamination was very similar in the output water of the units undergoing periodic treatment with Rely+On^TM^ Peracilyse, carried out only at the end of the day (untreated *vs.* periodically treated: *p* > 0.05 for both HPCs at 37 and 22 °C). *Legionellae* were detected from dental units in nine different operating spaces (24.3%), all on the first floor.

**Table 4:** Microbial contamination values of water samples from supply water and dental unit water systems at baseline.

Parameters		Supply Water (Tap Water)	Dental Units			
			No Disinfection Treatment	Periodic Disinfection (Rely + On^TM^ Peracilyse)	Continuous Disinfection (ICX^®^ 0.01%)	Continuous Disinfection (Calbenium^®^ 2%)
		n: 9	n: 11	n: 37	n: 11	n: 4
Temperature						
mean (°C)		17.9	24.9	23.3	24.5	26.3
SD (°C)		2.1	2.2	3.3	1.6	2.6
HPC 37 °C						
not compliant samples (%)		0	100	75.6	18.2	25.0
geometric mean (cfu/mL)		5.3	519.3	202.3	7.1	9.9
range (cfu/mL)		(1–20)	(55–4800)	(1–8720)	(1–221)	(1–236)
HPC 22 °C						
not compliant samples (%)		0	36.4	45.9	18.2	25.0
geometric mean (cfu/mL)		9.3	62.3	68.8	7.8	26.6
range (cfu/mL)		(2–98)	(14–634)	(1–5160)	(1–236)	(4–251)
*P. aeruginosa*						
positive samples (%)		0	9.1	27.0	0	75.0
range of positive samples (cfu/100 mL)			(75)	(100–3700)		(2–1020)
*L. pneumophila*						
positive samples (%)		22.2	18.2	13.5	63.6	50.0
range of positive samples (cfu/L)		(450–1250)	(200–300)	(350–3050)	(50–9000)	(250–1750)
Other species of *Legionella*						
positive samples (%)		0	18.2	10.8	0	0
range of positive samples (cfu/L)			(300–1100)	(50–250)		

The continuous disinfection systems, with both products (ICX^®^ and Calbenium^®^), achieved the lowest levels of HPC at 37 and 22 °C, with 80.0% of samples conforming to the limits of the ADA as well as those of the Italian norms for drinking water. The differences were statistically significant for both the HPC at 22 °C (untreated *vs* continuously treated *p* < 0.05; periodically treated *vs* continuously treated *p* < 0.05) and the HPC at 37 °C (untreated *vs* continuously treated *p* < 0.001; periodically treated *vs* continuously treated *p* < 0.001). However, the continuous disinfection methods were not totally effective against *L. pneumophila*, which was detected in 63.6% of samples treated with ICX^®^ and in 50% of sample treated with Calbenium^®^, all collected from the first floor of the main building. Of those treated with Calbenium^®^, three out of four were also contaminated by *P. aeruginosa* with counts up to 10^3^ cfu/100 mL, although much lower than the values considered to be the infective dose in healthy people (>1.5 × 10^6^ cfu/mL) [19].

These results show that the continuous introduction of disinfection products, necessarily used at low levels to minimize their potential toxic effect, was more effective in maintaining the heterotrophic bacterial counts within the recommended standards in output water of dental devices, while this treatment was not always able to control microorganisms such as *Legionella* and *P. aeruginosa*, which are very resistant to disinfectant treatments both on account of their intrinsic characteristics and because they are protected within the biofilm [41].

*L. pneumophila* was isolated from 75% of the units in the Surgery Division, 57.1% of those in the Periodontics Division, 50.0% in the Endodontrics Division, 42.8% in the Prosthesis Division and from 10.0% units in the Dental School Area. However, it was not possible to relate the contamination to the type of work carried out in the different divisions since the dental units of each division came from different manufacturers and underwent different treatments. Instead, the contamination from *Legionella* spp. appears to be related to the location of divisions in the various parts of the building. *Legionella* spp. were never detected in the operating spaces situated on the ground floor of the main building; the first floor of the main building was found to be colonized by *L. pneumophila* SG1 (54.2% of positive dental units); the ground floor of the secondary wing by *L. anisa* (18.2%), and the first floor of the secondary wing by *L. pneumophila* SG1 (10.8%) and *L. anisa* (10.0%).

The dental clinic is supplied from a single water system with several branches that distribute water from the public network to the various parts of the building. Over time, the water system has undergone modifications that may have led to dead legs and points of stagnant water in certain parts of the network but not in others. This may explain the different patterns of contamination found in dental units in relation to their location. Water temperature may also have affected the level of contamination: the temperature of samples taken from dental units of positive divisions was on average about 2 °C higher than those taken from negative divisions (24.0 °C *vs*. 22.2 °C), with statistically significant differences (*p* < 0.001).

The highest concentrations of *Legionella* spp. were detected on the first floor, in the dental units situated in the south-facing side of the main building (surgery and endodontrics), the same location as the positive samples of tap water. In these divisions 60% of the units were positive for *L. pneumophil*a SG1, at concentrations up to 9000 cfu/L of planktonic bacteria. According to the Italian guidelines for legionellosis prevention and control, concentrations above 10^3^ cfu/L are considered to represent a health hazard in healthcare settings and a sign that corrective measures need to be implemented [26].

Finally, no significant differences were found between the bacterial loads (HPCs at 37 and 22 °C) detected in samples positive or negative for *L. pneumophila*. The same was observed for *P. aeruginosa*.

### 3.2. Monitoring after the Adoption of the Risk Management Plan

To eliminate the contamination from *P. aeruginosa* and *L. pneumophila*, the units equipped with an independent supply system underwent shock treatment, by adding hydrogen peroxide 3% to the system. The shock procedure is described in Table 3. This treatment allowed *P. aeruginosa* and *Legionella* spp. to be eliminated and the HPCs to be reduced to levels lower than those prescribed for drinking water in Italy. Since shock treatments are known to be ineffective in the long term, the protocol described in Table 3 was adopted immediately afterwards, consulting the manufacturer about the products to be used in the different types of dental units. One of the units examined at baseline from the Castellini company and one from the Eurodent company had been replaced with two new units from A-dec. For technical reasons, periodic disinfection was not possible in the three remaining dental units from the Eurodent company, which were under continuous treatment with Calbenium^®^. Moreover, all the units with an independent supply system used deionised water.

After about a year from the baseline monitoring, the post-intervention monitoring produced the results reported in Table 5. The measures adopted allowed the contamination to be contained, with significant reductions in samples not conforming for HPCs. The results varied depending on the type of disinfection treatment implemented.

**Table 5:** Microbial contamination values of water samples from dental unit water systems after implementation of the risk management plan.

Parameters		Dental Units			
		No Disinfection Treatment	Disinfection Treatment (Supplied with Deionised Water)		
		Supplied with Tap Water	Continuous (H_2_0_2_ 0.06%) + Periodic (Rely + OnTM Peracilyse)	Continuous (ICX^®^ 0.01%) + Periodic (Sterilex Ultra)	Continuous (Calbenium^®^ 2%)
		n: 10	n: 37	n: 13	n: 3
Temperature					
mean (°C)		24.8	22.6	23.6	22.3
SD (°C)		2.2	2.1	1.0	1.1
HPC 37 °C					
not compliant samples (%)		100	35.1	38.5	33.3
geometric mean (cfu/mL)		874.9	17.1	9.4	13.0
range (cfu/mL)		(225–1980)	(1–121)	(2–100)	(3–21)
HPC 22 °C					
not compliant samples (%)		90.0	13.5	7.7	33.3
geometric mean (cfu/mL)		456.2	53.6	24.6	36.4
range (cfu/mL)		(77–1720)	(2–242)	(3–282)	(20–115)
*P. aeruginosa*					
positive samples (%)		20.0	0	0	0
range of positive samples (cfu/100 mL)		(240–300)			
*L. pneumophila*					
positive samples (%)		10.0	0	0	0
range of positive samples (cfu/L)		(250)			
Other species of *Legionella*					
positive samples (%)		10.0	0	0	0
range of positive samples (cfu/L)		(1850)			

The dental units which, for technical reasons, were not treated with the combined continuous and periodic disinfection protocol, showed an increase of HPCs (HPC 37 °C, *p* < 0.05; HPC 22 °C, *p* < 0.05). On the contrary, the HPCs decreased significantly in the output water from dental units treated with the combined H_2_O_2_ + periodic Peracilyse system (HPC 37 °C, *p* < 0.001; HPC 22 °C, *p* < 0.05) and remained at low concentrations (not significant differences pre-post) in dental units treated with continuous disinfection at baseline (ICX^®^ and Calbenium^®^) (Table 6).

*L. pneumophila* and *P. aeruginosa* were detected respectively in 10% (*vs* 18.2% at baseline) and 20% (*vs.* 18.2% at baseline) of the samples from untreated dental unit waterlines. The implementation of the combined continuous and periodic disinfection allowed *L. pneumophila* and *P. aeruginosa* to be completely removed from dental unit waterlines treated with H_2_O_2_ + Peracilyse and ICX + Sterilex ultra systems. The complete removal of these bacteria was also obtained in the dental units which continued to be treated only with Calbenium^®^ (Table 5). This result could be attributable to the implementation of management measures other than the disinfection methods, and suggests that all the combined interventions foreseen in the water safety plan contribute to the control of contamination and none of them should be overlooked.

**Table 6:** Comparison of the bacterial contamination of output water from dental units before and after implementation of the risk management plan.

Parameters		Dental Units Grouped for Disinfection Treatment								
		No Disinfection Treatment		Continuous (H_2_0_2_ 0.06%) + periodic (Rely+OnTM Peracilyse)		Continuous (ICX^®^ 0.01%) + Periodic (Sterilex Ultra)		Continuous (Calbenium^®^ 2%)		
		n: 10		n: 35		n: 11		n: 4	
		before	after	before	after	before	after	before	after
HPC 37 °C									
geometric mean (cfu/mL)		519.3	874.9	202.3	17.1	7.1	9.4	9.9	13.0
pre-post comparison (paired t test)		*p* < 0.05		*p* < 0.001		ns		ns			
HPC 22 °C									
geometric mean (cfu/mL)		62.3	456.2	68.8	53.6	7.8	24.6	26.6	36.4
pre-post comparison (paired t test)		*p* < 0.01		*p* < 0.05		ns		ns	

Notes: ns: not significant.

The combination of a continuous introduction of low levels of a minimally toxic agent associated with a periodic treatment using a more concentrated active product, has shown itself to be a potential method for the control of contamination. In addition, the periodic treatment may be useful in preventing the adaptive resistance of bacteria that could be induced by continuous exposure to low concentrations of biocides [42]. The combined procedure is easy to perform and therefore favours staff compliance to the protocol. Under our working conditions, we used various active products for dental units made by different manufacturers and obtained similar reductions in the microbial contamination, thus confirming that the efficacy is not so much linked to the product used, but rather to the type of treatment protocol applied and, above all, to the compliance of personnel to the management plan [3]. The literature reports that non-compliance and technical errors are the most probable causes of failure to properly disinfect dental unit waterlines [3,5].

This study was carried out in a complex clinical dental setting where various confounding factors may have affected the results, such as the age and technical characteristics of the dental units, their different activity and frequency of use, the various disinfection systems, and the location of the operating spaces. Despite this limitation, the results highlight the importance of identifying and implementing a management protocol for *Legionella* control, including microbiological monitoring focused on *Legionella* spp.

## 4. Conclusions

The following conclusions can be drawn from this study:

(1) The detection of bacterial loads (HPCs) below the recommended limits does not guarantee that dental unit waterlines are not contaminated by potentially pathogenic bacteria such as *L. pneumophila* and *P. aeruginosa*, thus confirming the results of Aprea *et al.* [43] and Bristela *et al.* [44]. Therefore, besides the technical-practical measures and disinfection protocols, an integrated approach for microbial risk management in a dental health care setting should also include the monitoring of these bacteria on a regular basis, in agreement with Pasquarella *et al.* [11].
(2) In order to control the contamination of dental units, an internal control plan is necessary. The adopted control measures, including the combined continuous and periodic water disinfection, are effective in the control of *Legionella* contamination. Environmental surveillance for *Legionella* is useful not only to assess the efficacy of preventive measures, but also as a guide for the choice of corrective strategies, in accordance with the principles of the internal control plan.
(3) In this study, the primary source of *Legionella* species was the water used to supply dental units. Therefore, the control of *Legionella* in dental health care settings also involves stakeholders other than dental staff. In particular, the domestic water providers should guarantee that the water distributed to the users is free from pathogenic bacteria such as *Legionella* spp. In addition, the manufacturers of dental chairs should equip dental units with independent supply water systems and disinfection methods.
(4) Dentists and other dental operators, technical staff, microbiologists, and public health professionals should work towards the common aim of guaranteeing the safety of patients and personnel. The collaboration of the manufacturers of the dental units is also essential to determine the best equipment and method for maintaining and monitoring good water quality and not to expose the dental units to treatment agents which could damage some of their components [27]. For this reason, the risk management plan lays special stress on the training of dental health workers and technical staff who must respect the good practices in operation and strictly adhere to protocols.

## Supplementary Files

Supplementary File 1
