# Supplementary figures and images for: Impact of a Risk Management Plan on Legionella Contamination of Dental Unit Water
Source: Int J Environ Res Public Health. 2015 Feb 23;12(3):2344–58. doi: 10.3390/ijerph120302344 (PMC4377905; doi:10.3390/ijerph120302344)

# Map of the dental clinic

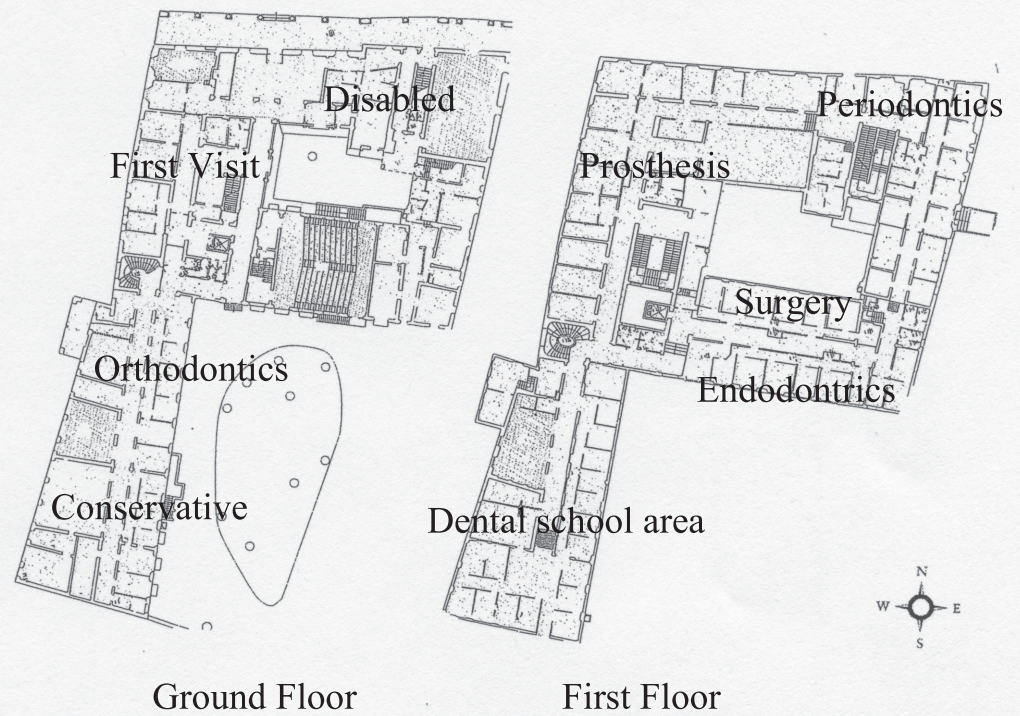

Supplement: Supplementary File 1 [file ijerph-12-02344-s001.pdf]
